# Polymer-Based Delivery of Glucagon-Like Peptide-1 for the Treatment of Diabetes

**DOI:** 10.5402/2012/340632

**Published:** 2012-05-30

**Authors:** Pyung-Hwan Kim, Sung Wan Kim

**Affiliations:** Center for Controlled Chemical Delivery, Department of Pharmaceutics and Pharmaceutical Chemistry, University of Utah, Salt Lake City, UT 84112, USA

## Abstract

The incretin hormones, glucagon-like peptide-1 (GLP-1) and its receptor agonist (exendin-4), are well known for glucose homeostasis, insulinotropic effect, and effects on weight loss and food intake. However, due to the rapid degradation of GLP-1 by dipeptidylpeptidase-IV (DPP-IV) enzyme and renal elimination of exendin-4, their clinical applications have been restricted. Although exendin-4 has longer half-life than GLP-1, it still requires frequent injections to maintain efficacy for the treatment of diabetes. In recent decades, various polymeric delivery systems have been developed for the delivery of GLP-1 and exendin-4 genes or peptides for their long-term action and the extra production in ectopic tissues. Herein, we discuss the modification of the expression cassettes and peptides for long-term production and secretion of the native peptides. In addition, the characteristics of nonviral or viral system used for a delivery of a modified GLP-1 or exendin-4 are described. Furthermore, recent efforts to improve the biological half-life of GLP-1 or exendin-4 peptide via chemical conjugation with various smart polymers via chemical conjugation compared with native peptide are discussed.

## 1. Introduction

Diabetes is classified into two types, depending on the insulin production in body [1]. One is type 1 diabetes, which type 1 diabetes accounts for 5% to 10% of all diabetes cases in the USA. In type 1 diabetics, insulin is not produced, due to destruction of the pancreatic *β*-cells, by autoimmune response. The other is type 2 diabetes and this constitutes 90% to 95% in all diabetes cases. The features of type 2 diabetes are characterized by insulin resistance, hyperglucagonaemia, pancreatic *β*-cell dysfunction, and abnormally high rate of *β*-cell apoptosis [2, 3]. For the treatment of diabetes, incretin hormones such as glucagon-like peptide-1 (GLP-1) and GLP-1 receptor agonist (exendin-4) have been investigated because they act in opposition against the characterizations of diabetes [4]. There are a large number of papers reviewed with respect to action and effects of GLP-1 and exendin-4 action of effects [5–7].

**Figure 1:**
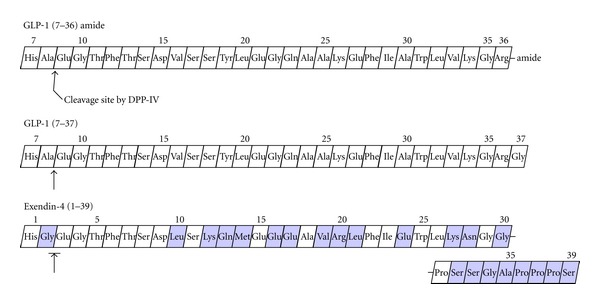
Amino acid sequence of GLP-1 and exendin-4. GLP-1 consists of two active circulating forms, GLP-1 (7–36) amide and GLP-1 (7–37).

The biological function of GLP-1 is well known as an incretin hormone released from gastrointestinal L-cells [1, 3]. Native GLP-1 (1–37) is produced two active circulating forms, GLP-1 (7–37) and GLP-1 (7–36) amide [6] (Figure 1). GLP-1 (7–36) amide is more abundant in blood. Incretin effect of GLP-1 is reviewed in several papers [2, 3, 6]. Intact GLP-1 promotes insulin secretion, inhibits glucagon secretion in pancreas, and increases the synthesis of proinsulin. Furthermore, GLP-1 promotes proliferation of pancreatic islet *β*-cell, inhibits *β*-cell apoptosis, delays in gastric emptying, and induces weight loss by suppression of appetite in type 2 diabetes [2, 5]. GLP-1, also, enhances the hepatic glucose uptake due to increasing glycogen syntheses activity [8]. Although GLP-1 has many advantages, however, its application in clinical trials is considerably restricted because GLP-1 is degraded rapidly by dipeptidyl-peptidase (DPP-IV), along with renal clearance [9]. Accordingly, long-acting GLP-1 receptor agonists such as exendin-4, which are more resistant to degradation by DPP-IV, may have a long term therapeutic efficacy compared with GLP-1 [2].

GLP-1 receptor agonist, exendin-4, is an exocrine hormone isolated from the parotid gland of the Gila monster lizard. Exendin-4 has, also, been shown to inhibit glucagon secretion, reduce glucose clearance, stimulate insulin secretion, protect against *β*-cell apoptosis, promote *β*-cell proliferation, promote satiety, and inhibit gastric emptying [10, 11]. It was reported that exendin-4 binds to GLP-1 receptor, since exendin-4 has a 53% amino acid sequence homology of GLP-1 [12] (Figure 1). According to report that GLP-1 receptors are detected in multiple cells within the immune system [13], GLP-1 or exendin-4 collectively suggests that current incretin therapies may have multiple beneficial effects in patients with type 1 diabetes, including improved glycemic control via multiple mechanisms. Furthermore, exendin-4 was also used as a target moiety for *in vivo* pancreatic islet imaging [14].

In addition, the GLP-1 receptors are present on various tissues [6]. It is also expressed in cardiac myocytes [15]. Preclinical and clinical studies have shown that GLP-1 and exendin-4 are strongly linked to salutary cardiovascular effects [16, 17]. This supports the facts that cardiovascular and cerebrovascular diseases account for around 65% of mortality among patients with diabetes [18–20]. Several studies demonstrated that the symptoms of cardiovascular diseases were improved during treatment with GLP-1 or exendin-4 therapy and that GLP-1 protected heart against cardiac remodeling after myocardial infarction [18, 21, 22]. Indeed, GLP-1 receptor in the brain mediates not only the prevention of neuronal degeneration by GLP-1, but the enhancement of central nervous system cell survival and function by exendin-4 [10].

In spite of these advantages of GLP-1 or exenedin-4, however, its clinical trials are still restricted because GLP-1 has very short *in vivo* half-life (<2 min). In addition, administration of exendin-4, which has longer half-life than GLP-1 produced anti-exendin-4 antibodies, resulting in the appearance of diminished glycemic response and the requirement of readministration [3].

New strategies should be developed for an effective long-term action of GLP-1 or exendin-4. Several approaches have been undertaken to develop agents that replicate or replace the actions of GLP-1. Some of them are in various stages of clinical development [23, 24]. One way among several innovative methods is to deliver exogenous incretin hormone genes or peptides using polymeric carriers. Currently, delivery carriers are classified two parts: non-viral and viral system. Nonviral vector using liposomes, naked DNA, and synthetic polymers has many advantages such as low cytotoxicity, low immunogenicity, and unlimited size for transgene. On the other hand, viral vectors using viruses has advantages such as high and stable intracellular delivery. With respect to these delivery carriers, it will be introduced in below each section.

In this paper, we will describe with a focus on intriguing vector designs, potentials of the techniques for long-lasting action, ectopic production, and secretion of GLP-1 or exendin-4 via a variety of polymers, which are classified with characteristics using nonviral system, viral system, and modified peptides based on polymeric delivery.

## 2. Delivery and Therapeutic Systems on Effects of GLP-1 and Its Agonist

Current approaches for the delivery of GLP-1 and exendin-4 for higher and longer therapeutic effects will be discussed with the introduction of various polymers. One approach is to deliver the DNA coding GLP-1 or exendin-4. The DNA will produce therapeutic proteins for a prolonged time, suggesting that gene delivery may overcome shortcomings of the peptide delivery. Another approach is to modify the peptides with polymers to inhibit or reduce enzymatic degradation of the peptides.

### 2.1. NonViral Delivery Carriers for Gene Expression System

NonVirall gene delivery carriers have some advantages compared with viral gene delivery carriers, such as lower immunogenicity, highly reproduction, and a simple quality control process, excellent safety profile, and ability to carry large amounts of DNA [25]. One part among a variety of nonVirall carriers including liposomes and polymers is to exploit the natural ability of cationic polymer. Cationic polymers condense plasmid DNA (pDNA) by electrostatic interaction, forming polyplexes [26]. In this section, current incretin gene expression systems and polymeric carriers for the incretin genes expression systems are discussed (Table 1).

#### 2.1.1. Poly(ethylenimine) (PEI)

PEI has high charge density, composed of 25% primary amines, 50% secondary amines, and 25% tertiaryamines [27]. It has been shown to effectively condense pDNA into colloidal particles that effectively transfect DNA into a variety of cells both *in vitro* and *in vivo*. Currently, branched 25 kDa PEI has been widely used for gene delivery due to transfection efficiency and lower cytotoxicity than higher molecular weight PEI [28, 29].

PEI has been used for delivery of the GLP-1 constructs. The first GLP-1 plasmid was composed of the modified GLP-1 cDNA with furin cleavage site. The transcription of the GLP-1 cDNA was driven by chicken *β*-actin enhancer/promoter (p*β*-GLP-1), which has been known as one of the strongest mammalian promoters [30]. The first two amino acids are the receptor binding site. For the translation initiation, the first codon for the GLP-1 cDNA was methionine. However, the first methionine might interfere with the interaction between GLP-1 and its receptor by masking the first two amino acids. Therefore, the methionine should be removed after translation for full activity. In this approach, we designed the GLP-1 plasmid with furin recognition site. After translation, the first methionine, coded by the initiation codon, was removed in Golgi apparatus, producing wild-type GLP-1. *In vitro* transfection into HepG2 cells and coculture assays with rat islets showed that the produced GLP-1 had insulinotropic effect. Zucker diabetic fatty (ZDF) rats were treated with PEI/p*β*-GLP-1 polyplex via intravenously single injection. The results showed therapeutic effects by increasing insulin and reducing blood glucose levels for 2 weeks. However, the therapeutic effects were not enough to normalize the blood glucose level.

To increase the therapeutic effects of the GLP-1 gene therapy, two approaches were employed. The first was to improve the expression plasmids by employing the NF*κ*B binding site. The modified GLP-1 plasmid vector (pSIGLP-1/NF*κ*B) with 5x nuclear factor *κ*B (NF*κ*B) binding sites was constructed to enhance nuclear import of the plasmid [31]. NF*κ*B is a transcription factor, which is produced in cytoplasm. Without any activation signal, NF*κ*B resides in cytoplasm as a complex with I*κ*B. However, on activation, NF*κ*B translocates into the nucleus. The activation signals include innate immune response such as activation of toll-like receptors (TLRs) and tumor formation phobal esters. However, without these signals, basal level NF*κ*B translocates into the nucleus continuously. In this nuclear translocation process, NF*κ*B may recognize its binding sites of the GLP-1 expression plasmids and cotranslocates with the plasmid into the nucleus. Therefore, it may increase nuclear trafficking of the transfected plasmid and gene expression. This approach is not limited to GLP-1 gene therapy and may be useful for various gene therapies. Diet-induced obese (DIO) mice were treated with PEI/pSIGLP-1/NF*κ*B complex. The results showed that the blood glucose levels were decreased and maintained for 3 weeks. In addition, the plasma insulin levels increased in a similar way, while weight gain and food intake were reduced.

Another approach to improve the GLP-1 expression plasmid was to employ the two-step transcription amplification systems (TSTA). The first TSTA system for the GLP-1 expression was constructed and evaluated *in vitro* by Lee et al. [32]. This system was composed of the two expression plasmids. In the first plasmid, the Gal4-DNA binding domain (DBD)/p65-transactivation domain (TAD) fusion protein was expressed under the control of the chicken *β*-actin enhancer/promoter. Then, the expressed Gal4-DBD/p65-TAD protein binds to the upstream activating sequence (UAS) in the second plasmid and then stimulated the transcription of the GLP-1 gene. It is important to determine the optimal ratio between first and second plasmids for a high expression of GLP-1. In their *in vitro* data, the TSTA system induced the GLP-1 expression by 4-fold compared with p*β*-GLP-1 system. In this *in vitro* evaluation, the TSTA system was delivered with PEI. For optimum expression, two plasmids should be delivered into a same cell. The complex of PEI and the TSTA system may have more than one plasmid, and some complexes might have both p*β*-Gal4-p65 and pUAS-GLP-1 for TSTA effect. However, it is unlikely that all complexes have two plasmids. Therefore, it may be useful to construct the GLP-1 TSTA system in a plasmid, which has both Gal4-p65 and UAS-GLP-1 expression units.

#### 2.1.2. Arginine-Grafted Bioreducible Polymer (ABP)

PEI has significant acute toxicity concerns such as cellular toxicity, aggregation of erythrocytes, and entrapment in the lung capillary [51], and the application of PEI to clinical settings is strictly limited. Therefore, a safe and efficient carrier should be developed for GLP-1 gene therapy. One of the important approaches is to develop biodegradable gene carriers. There are several examples of biodegradable gene carriers. For example, poly[*α*-(4-aminobutyl)-L-glycolic acid] (PAGA) is a degradable gene carrier with ester bonds in its backbone. PAGA was used for delivery of interleukin genes for diabetes [52] or cancer gene therapies [53]. PAGA was nontoxic and did not induce any cytotoxicity *in vitro* and *in vivo*, suggesting that biodegradability is one of the important requirements for safe gene therapy. However, ester bonds in PAGA were rapidly degraded in aqueous solution and PAGA had relatively short half-life. Therefore, another approach was employed for the development of biodegradable gene carriers. Disulfide bonds are stable in extracellular space, while they are rapidly degraded in reduction condition in cytoplasm. Therefore, the carrier with disulfide bonds forms stable complex with pDNA outside of cells but releases pDNA rapidly inside of cells after entry to cells. According to this approach, arginine-grafted bioreducible polymer (ABP) was synthesized and evaluated as a gene carrier for the GLP-1 gene. ABP had higher transfection efficiency than PEI in various types of cells and tissues *in vitro* and *in vivo* and did not induce any significant toxicity to cells [33, 54–57].

ABP was evaluated for delivery of a novel TSTA-GLP-1 expression system with the secretion signal peptide (SP). The TSTA-GLP-1 expression system based with SP was constructed by insertion of SP at the upstream of the GLP-1 cDNA of pUAS-GLP-1 [58]. Therefore, the TSTA system was composed of p*β*-Gal4-p65 and pUAS-SP-GLP-1. The TSTA-GLP-1 system with SP (TSTA-SP-GLP-1) was complexed with ABP and then delivered into cells *in vitro*. The ABP/TSTA-SP-GLP-1 polyplex increased the GLP-1 expression levels and improved the insulin secretion-inducing ability *in vitro*. *In vivo* study may be required for fully identification of the effects of TSTA-SP-GLP-1 with ABP polymer.

The most efficacies among our previous results about the treatment of diabetes were showed in experiment of exendin-4 expressed by TSTA system with secretion signal sequence (unpublished data). In this study, TSTA-SP-exendin-4 with ABP polymer (ABP/TSTA-SP-exendin-4) led to the antidiabetic efficacies for 12 days without hypoglycemia in DIO mice.

#### 2.1.3. Chitosan

Chitosan is safe, nontoxic, cationic, and biodegradable polysaccharides, composed of D-glucosamine and N-acetyl-glucosamine [26, 34, 59]. Chitosan was first used as a carrier for the delivery GLP-1 gene for the treatment of diabetes by Jean et al. [60]. In this study, they constructed a GLP-1 plasmid (pVax1-GLP-1) which contained a furin recognition site to ensure posttranslational processing bearing an N-terminal histidine residue (His^7^) in pVax1 vector for effective gene expression. This approach using the furin recognition site was exactly the same as the previous approach to remove the initiation methionine from the produced GLP-1 peptide. The expression of GLP-1 was driven by the cytomegalovirus immediate early (CMV IE) promoter and enhancer. The therapeutic effect of the most effective chitosan-based complex (chitosan 92-10-5; degree of deacetylation or DDA-MW in kDa-chitosan/DNA N : P ratio) containing pVax1-GLP-1 was examined by two routes of 7 times administration, intramuscular (i.m.), and subcutaneous (s.c.), at days 0, 7, 14, 21, 35, 49, and 63 in the ZDF rat. The highest plasma GLP-1 levels were observed in mice treated with chitosan/pVax1-GLP-1 complex compared with those of pVax1-GLP-1 via both s.c. and i.m. in 14 days after final treatment (day 77). At the same time, insulin levels increased more than 2-fold compared with that of uncomplexed plasmid. This increase in the insulin level coincided with a sustained GLP-1 expression in plasma. Mice treated with chitosan/pVax1-GLP-1 complex, also, induced a reduction in food intake and body weight gain. In conclusion, the incretin effects of pVax1-GLP-1 with chitosan maintained for 14 days following the last injection. However, the CMV promoter and enhancer have silencing effect. The methylated CpG motifs induce innate immune response and increase the secretion of proinflammatory cytokines [61]. The increased cytokines may result in the hypermethylation of the CMV promoter and enhancer, decreasing promoter activity. Therefore, the mammalian promoters such as *β*-actin promoter may be a better selection than the CMV promoter.

#### 2.1.4. Intramuscular Injection and Electroporation of Naked Chimeric GLP-1/IgG-Fc Plasmid

Electroporation is a widely used technique for the transfection of DNA *in vitro* and *in vivo*. This can, also, be used to introduce DNA into muscle, skin, liver, and cancer cells as well as to control localization of transgene expression by DNA injection site [35].

Kumar et al. constructed a plasmid that expressed a fusion protein of human GLP-1 and mouse IgG heavy chain constant region with the Ig *κ* secretion leader peptide [62]. Conjugation of IgG-Fc region prolongs circulation time of many peptide drugs [63, 64]. The expressed GLP-1/IgG-Fc fusion protein showed that it was capable of activating GLP-1R via the cAMP induction and stimulating insulin secretion from islet *β*-cells in a glucose-dependent manner *in vitro*. In the results to examine *in vivo* effect of GLP-1/IgG-Fc protein, diabetic db/db mice were intramuscularly injected twice (2 weeks interval after first administration) with GLP-1-IgG-Fc/VRnew or IgG-Fc/VRnew (control vector) plasmids, and then the muscles were electroporated using electrodes. Although the *in vivo* effects of GLP-1/IgG-Fc fusion protein on lowering blood glucose levels was not interestingly different between the two groups of mice, the fasting blood glucose levels were shown 1.7-fold lower than the control mice at 12 weeks after first injection. Moreover, the fasting insulin and glucagon levels showed about 1.6-fold-enhanced insulin secretion and 1.3-fold-reduced glucagon release in mice treated with GLP-1/IgG-Fc plasmid compared with the IgG-Fc-treated control mice. This result showed that GLP-1/IgG-Fc system could apply long-lasting effects of GLP-1 for the treatment of diabetes.

### 2.2. Viral Delivery Carriers for Gene Expression System

Viral vectors have been used to deliver the GLP-1 gene to target cells. Viral vectors have high levels of cellular transfection, high intracellular delivery by easy escape from the endosome, nuclear import, and transcriptional stability, compared with nonvirall vector system [55, 57, 65]. In this part, we described about GLP-1 or its mimetic gene delivery using viral vectors including adenovirus (Ad) or adeno-associated virus (AAV) (Table 2).

#### 2.2.1. GLP-1 and Exendin-4 Gene Delivery Using Adenovirus

Adenovirus is one of the most potent gene delivery vectors used in gene therapy. Also, Ad has widely been used for clinical application, due to their advantages. First, they are produced with high titer and high transduction efficiency in dividing and nondividing cells. Second, they do not induce the mutagenesis by insertion of their genome into the host DNA. Furthermore, their biological characteristics of virus are well known [66, 67]. It is, however, well known that they are accumulated in the liver and induce immune system after systemic administration [36, 65, 68]. Many GLP-1 delivery studies using Ad have been performed with various expression cassette designs of GLP-1 gene rather than vector itself modification for long-term expression and secretion of active GLP-1 in ectopic tissues.

Parsons and colleagues incorporated a novel GLP-1 expression cassette in adenoviral gene to express exogenous GLP-1 [69]. They constructed the GLP-1 mini-gene mutated with Ala at position 8 to Gly. The CMV enhancer and ubiquitin promoter were used for the GLP-1 expression. The exendin-4 leader sequence was located at the upstream of the GLP-1 gene for secretion of GLP-1 after expression (pCUbiEX4GLP-1Gly8). The CUbi vector with the CMV enhancer and ubiquitin promoter is well known for directing expression for more than 1 month in the liver [37]. Injection of Ad (Ad-CUbiEX4GLP-1Gly8) containing this GLP-1 expression cassette showed the improved glucose homeostasis after single intravenous (i.v.) injection of 1 × 10^10^ or 1 × 10^12^ viral particles (VP) of Ad in db/db mice or ZDF rats, respectively. These euglycemic effects maintained for 6 weeks of the study, whereas plasma insulin levels showed no significant difference between control groups.

Another GLP-1 expression system using adenoviral vector was constructed by Jun's group [70]. They used expression system contained CMV promoter, *β*-globin/IgG chimeric intron, the GLP-1 cDNA, and albumin leader sequence. In their first report, streptozotocin- (STZ-) induced nonobese diabetic/severe combined immunodeficiency (NOD/SCID) mice or spontaneously diabetic NOD mice were intravenously injected one time with 6 × 10^11^ VP or 1 × 10^12^ VP of Ad-GLP-1, respectively. NOD/SCID mice treated with Ad-CMV-GLP-1 showed normoglycemia for 30 days postadministration, while serum insulin levels were observed higher in mice treated with Ad-GLP-1 compared with mice treated with control Ad. In addition, NOD mice treated with CFA, which prevent autoimmune response agent *β*-cell, and Ad-CMV-GLP-1 maintained lowering blood glucose levels for 1 year due to the inhibition of autoimmune attack of regenerated *β*-cell. In another study, single administration of Ad-CMV-GLP-1 in ob/ob mice resulted in antidiabetic effects such as the improvement of *β*-cell function, reduction of gluconeogenesis, and the improvement of insulin sensitivity [38].

Antidiabetic effects of exendin-4 expressed by Ad were reported by Samson and colleagues [39]. They used a helper-dependent adenovirus (HDAd) vector with exendin-4 driven by the CMV promoter. The vector also contains a leader sequence for enhanced secretion and the furin cleavage for production of wild-type exendin-4. Single injection (i.v.) of HDAd-CMV-exendin-4 (1 × 10^11^ VP) in diet-induced obesity (DIO) reduced the blood glucose levels and hepatic lipid. Exendin-4 was expressed for 15 weeks but did not induce the plasma insulin level, suggesting that the enhanced expression of exendin-4 may improve the insulin sensitivity.

In another study with Ad, Lee et al. constructed a GLP-1 expression vector with the CMV promoter and the insulin leader sequence followed by GLP-1 [71]. This expression cassette was incorporated in Ad or AAV vector (Ad-IL-GLP-1 or AAV-IL-GLP-1). The results suggest that the expression of GLP-1 induced insulin in dose- or glucose-dependent manner *in vitro*. *In vivo* animal study showed that administration of Ad-IL-GLP-1 achieved longed-glucose homeostasis and improved insulin sensitivity in ZDF rats treated with 2 × 10^12^ VP for 21 days.

Delivery of the GLP-1 or exendin-4 gene using viral vectors showed promising results of the applications of genes to diabetes. Disadvantage of Ad such as liver accumulation can rather work as an advantage for the production of incretin in hepatocyte. However, side-effects and toxicity of viral vectors may still have limited their application to clinics. Currently, viral vectors are useful for proof-of-concept rather than for clinical applications. It is more important to develop nontoxic viral vectors with little side-effects.

#### 2.2.2. GLP-1 Gene Delivery Using Adeno-Associated Virus

Unlike Ad, AAV is capable of integration into the human genome at a specific site on chromosome-19 [72, 73] and infectious to nondividing cells. In addition, it does not induce immune response of transduced cells, due to lack of viral genes. Therefore, AAV can achieve efficient and long-term gene transfer in a variety of tissues [40]. Especially, continuous and long-term control of insulin and glucose level is important. Unlike episomal vectors, AAV has higher rate of integration of their DNA into the host cell chromosome. However, the integration rate is not as high as retroviral vectors and further improvement of the integration rate may be beneficial for this purpose. Although there are integration hotspots in the human genome for AAV, some integration may induce oncological recombination of the genes. Therefore, integration of the viral genome into the host chromosome should be carefully controlled.

For the treatment of type 1 diabetes, AAV-MIP-GLP-1 produced GLP-1 gene by the mouse insulin-II promoter (MIP). MIP confined the gene expression specifically in pancreatic *β*-cells in a glucose-dependent manner. The proglucagon signal sequence was used for the facilitated secretion of the peptide [41]. In this study, i.p. injection of 1 × 10^12^ VP of AAV containing GLP-1 expression cassette increased the *β*-cell proliferation and maintained glucose homeostasis in the STZ-induced type 1 diabetes mice.

Recently, another study showed that normal glucose level was maintained for 4 months after portal-vein injection (1 × 10^12^ VP) of double-strand (ds) AAV containing a GLP-1 plasmid (dsAAV-CB-GLP-1) in db/db obese mice, suggesting that administration route may be an important factor for the success of the gene therapy. In this study, GLP-1 expression cassette in dsAAV was constructed with the CMV enhancer and chicken *β*-actin promoter (CB promoter), Ig *κ*-chain leader sequence, and a furin protease recognition sequence [42].

### 2.3. Peptide Delivery of Modified GLP-1 or Exendin-4 with Polymers

Although GLP-1 or GLP-1 receptor agonist have many incretin effects for diabetes, its short half-life *in vivo *still remain a question to overcome. Consequently, delivery of GLP-1 peptide *in vivo *has been focused on maintaining long-acting antidiabetic effects as well as resistance to degradation by DPP-IV enzyme. In order to maintain long-acting activity of peptide from degradation by DPP-IV enzyme, several kinds of polymers have been used with modified GLP-1 or exendin-4 peptide (Table 3).

#### 2.3.1. Sustained Release by Encapsulation of GLP-1 or Exendin-4

**(1) Controlled Release of GLP-1 Peptide Using Triblock Copolymer of PLGA-PEG-PLGA (ReGel)**

Long-term delivery of active GLP-1 is required for the treatment of diabetes by peptide delivery. Therefore, controlled release of GLP-1 from a depot is a desirable approach. ABA-type triblock copolymer, poly[(DL-lactide-co-glycolide)-b-ethylene glycol-b-(DL-lactide-coglycolide)] (PLGA-PEG-PLGA; ReGel), was used as a depot for controlled release of GLP-1 [43]. Regel is biodegradable gel in aqueous solution and performed sol-gel transition depending on temperature. Therefore, the mixture of GLP-1 and ReGel at room temperature was injected into animal model subcutaneously. At the body temperature, Regel formed gel immediately after injection and released GLP-1 gradually. ReGel released GLP-1 for over 14 days in ZDF rates after subcutaneous injection. Moreover, normal blood glucose level was maintained in rats injected with ZnGLP-1/ReGel compared with that of control rats for more than 14 days. Therefore, ReGel is a convenient and useful carrier for the GLP-1 peptide. ReGel formulation technique may provide normalization of blood glucose levels by control of loaded amount of GLP-1 and gel concentration [74].

**(2) Microencapsulation of GLP-1/Polymer Conjugate and Rat Islets**

For long-term therapeutic effect of GLP-1, poly(N-vinylpyrrolidone-co-acrylic acid-g-PEG) (VAP-) conjugated GLP-1 (VAPG), was microencapsulated in alginate with rat islets [44]. Zinc complexation of GLP-1 (GLP-1/Zn^2+^) was used as control of VAPG [75]. An *in vitro *study showed that microencapsulated islets with VAPG induced higher insulin secretion in a glucose-dependent manner, compared with the control group. Microencapsulated islets with VAPG, also, maintained the insulin secretion for 5 weeks, indicating that VAPG was efficient in promoting the functionality of encapsulated islets for a prolonged time. This prolonged effect of encapsulated islets may be due to antiapoptotic effect and *β*-cell proliferation effect of GLP1. This system could be reloaded with fresh islets and each component, suggesting that it would be served as a potential tool for long-term insulinotropic effect via adjustment of implanted islets.

**(3) Exendin-4 Inhalation Delivery System Adsorbed to Porous Large PLGA Microspheres**

Although exendin-4 has many therapeutic benefits for diabetes, its half-life is still short to fully treat diabetes mellitus. To induce sustained release and extended *in vivo* half-life exendin-4, modified exendin-4 adsorbed to improved porouspoly(lactic-co-glycolic acid) (PLGA) microparticle was produced by Kim et al., for antidiabetic inhalation [76]. PLGA-based biodegradable microparticles have been investigated for sustained and targeted/localized delivery of agents such as purified protein, bacteria, DNA, or viruses [26, 77].

In this study, exendin-4 was modified with the sixteen carbons of palmitic acid (palmityl-acylated exendin-4; Ex4-C_16_) for strong adsorption onto the hydrophobic surfaces of porous PLGA microspheres because of inducing the rapid release of therapeutic drugs by considerable empty volume, thin matrix frame of porous PLGA. Palmityl acylation to induce binding to human serum albumin, also, helps release of exendin-4 from PLGA microparticle to extend half-life of exendin-4. Adsorption onto and release from porous PLGA microparticle were confirmed by fluorescent dye conjugated to Ex4-C_16_. Specially, for the pulmonary delivery, the porous PLGA microparticle was directly administered into the lungs via trachea of mice. In the evaluation of the pulmonary hypoglycemic efficacy, glucose level in PLGA/Ex4-C_16_-treated mice was 2.6-fold lower than those of porous PLGA for the whole experimental time (150 h). This glucoregulatory effect continued until 5 days after the administration. Therefore, porous PLGA microparticle via inhalation delivery has pharmaceutical potential using lung deposition for antidiabetic peptides.

#### 2.3.2. PEGylation and Albumin Conjugates of GLP-1 or Exendin-4

**(1) PEGylation of GLP-1**

Polyethylene glycol (PEG) is an uncharged, hydrophilic, widely used polymer. PEGylation can reduce protein-protein interaction, resulting in circulation time of therapeutic proteins [26, 45]. PEGylation of a protein extends *in vivo* pharmacokinetics by increasing the clearance time in the blood, decreases proteolytic degradation, and reduces immune response [78].

Biological activity of PEGylated GLP-1 was first investivated by Lee et al. [79]. The PEGylation sites were with site-specific PEG_2k_ conjugation at position His^7^ in N-terminal by andaldehyde monomethoxypoly-PEG (mPEG-N-GLP-1) and Lys^26^/Lys^34^ in C-terminal by succinimidyl propionate monomethoxypoly-PEG (mPEG-Lys-GLP-1). In this study, PEGylated GLP-1 had the prolonged half-life and the improved proteolytic stability against DPP-IV enzyme, compared with native GLP-1. mPEG-Lys-GLP-1 increased insulin secretion more efficiently than mPEG-N-GLP-1 in rat pancreatic islets. This may be due to steric hindrance of mPEG-N-GLP-1. N-terminal amino acids of GLP-1 are the receptor binding sites and PEGylation of N-terminus may interfere with the interaction of the GLP-1 peptide and its receptor. Furthermore, mPEG-Lys-GLP-1 showed the improved plasma exposure and the stability, compared with native GLP-1 following s.c. or i.v. administration in Rats, suggesting the PEGylation effect. Actual antidiabetic efficacies of PEG-Lys-GLP-1 were assessed in db/db mice. The results showed that the administration of mPEG-Lys-GLP-1 reduced the blood glucose and increased the insulin levels [46].

Another example is PEGylated dual-acting peptide for diabetes (PEG-DAPD). Hjorth et al. reported that chimeric peptide consisting of the N-terminal of glucagon and the C-terminal of GLP-1 was recognized by both receptors with high affinity [47]. However, functional activity was not reported at that time. Recently, this dual-acting peptide for diabetes (DAPD) of GLP-1 receptor agonist and glucagon receptor antagonist was produced. This DAPD was reported to activate the GLP-1 receptor and inhibit the glucagon receptor [48]. Several site mutations were incorporated into the DAPD for enhanced therapeutic effects. PEGylation of DAPD was performed with 22 or 43 kDa PEG to overcome the short half-life of the peptides. PEG-PAPD was administrated into the rat models subcutaneously. The intraperitoneal glucose tolerance test (IPGTT) showed that PEG-DAPD reduced the blood glucose level, compared with control.

**(2) GLP-1-Albumin Conjugate (CJC-1131)**

As one of approaches to overcome the short half-life of GLP-1, Kim et al. modified the GLP-1 peptide with a single amino acid substitution of L-Ala^8^ to D-Ala^8^ at position 2 and a Lys^37^ addition to the C-terminus with selective attachment of a [2-[2-[2-maleimidopropionamido-(ethoxy)ethoxy]acetamide to the epsilon amino group of Lys^37^ [49]. The COOH-terminal end of this modified GLP-1 was conjugated to a short covalent reactive chemical linker and coupled to the specific cysteine residue in the albumin molecule for long half-life. This peptide was complexed with CJC-1131, producing the GLP-1-albumin drug affinity complex (DAC). For the evaluation of therapeutic effects, albumin/CJC-1131 was injected via various routes (i.p., s.c., or i.v.) in db/db mice following glucose loading. The results showed that i.p. injection of albumin/CJC-1131 reduced the glycemic excursion more efficiently, compared with that of s.c. injection. Intravenous CJC-1131 administration, also, produced a dose-dependent reduction in glycemic excursion following glucose loading. The prolonged repeated administration of albumin/CJC-1131 conjugate twice daily for 4 weeks reduced the fed blood glucose levels effectively compared with injection of saline. Albumin/CJC-1131 conjugate also stimulated islet cell proliferation and increased proinsulin mRNA transcripts, although an increase of plasma insulin was not observed in conjugate-treated mice.

**(3) Exendin-4 Conjugated with Human Serum Albumin via Hetero-Bifunctional PEG Linkage**

Compared with the direct conjugation to albumin, the conjugation of peptide and albumin was performed to exendin-4 with spacer of PEG by Kim's group. They reported that exendin-4 and albumin conjugates with longer PEG spacer increased the receptor binding affinity of exendin-4. This effect may be due to that the longer spacer the conjugates had, the less steric hindrance they had [50]. As long-space linker, a 5 kDa PEG was used for the conjugation of human serum albumin (HAS) and exendin-4. Finally, HAS-PEG-exendind-4 made via chemical conjugation showed 24.6- or 1.97-fold increased receptor binding affinity than Ex4 or HAS-exendin-4 in cell, respectively. Pharmacokinetics of HSA-PEG-exendin-4 in mice showed that PEG linker prolonged the half-life (24.4 h) compared with exendin-4 only (2.1 h) or HAS-exendin-4 (11.4 h) following i.p. administration. PEG linker also induced better hypoglycemic effects and prolonged duration compared with that of HSA-exendin-4 without a PEG linker in nonfasted diabetic mice. The results suggest that HAS-PEG-exendin-4 may be useful for treatment for type 2 diabetes with long-term duration of effect.

#### 2.3.3. GLP-1 or Exendin-4 Analogs Containing Disulfide Bond

Recently, Li's group introduced a modified GLP-1 peptide containing disulfide bond in wild-type GLP-1 for the treatment of type 2 diabetes. The presence of disulfide bonds in structure was suggested as an ideal tool to improve the stability of therapeutic peptide or protein. First, they constructed a modified GLP-1 homodimer by single mutation to cystein at position 10, 23, or 33 of wild-type GLP-1 [80]. As a result, among GLP-1 analogs, analog (hdGLP1G10C) mutated at position 10 of GLP-1 was the most biological active form, compared with native GLP-1. The rats treated with hdGLP1G10C showed an increased insulin secretion in 180 min following glucose loading. The hdGLP1G10C also showed long-acting glucoregulatory effects for 5 days by single-dose injection glucose tolerance in rats. In addition, ZDF rats treated with hdGLP1G10C had improved the diabetic condition, compared with GLP-1 after subcutaneously administration every 5 days during 35 days.

Li et al. also mutated GLP-1 or exendin-1 analogs with additional cysteine residue based on similar manner as described above. In addition, several glycine residues were attached at C-terminal tail to protect against the degradation by DPP-IV enzyme [81]. The most effective analog among the tested GLP-1 and exendin-4 analogs was GLP17057. The analog reduced the glucose levels, increased insulin levels, and showed long-lasting effects in rats, compared with wild-type GLP-1 or exendin-4. Therefore, interdisulfide bonds between GLP-1 would be a useful tool for improving the stability and therapeutic effects.

## 3. Conclusion

Incretin such as GLP-1 and exendin-4 has been the most important peptide drugs in diabetes research. There is no doubt that GLP-1 and its mimetric are attractive therapeutic materials for the diabetes treatment. However, short half-life has limited their application to clinics. Therefore, many research efforts have been made to overcome these problems. The approaches can be summarized into 4 categories. First, prolonged therapeutic effects are achieved by gene delivery with strong tissue-specific expression cassette to induce high production of incretin. However, the most difficult barrier in gene delivery is to develop an efficient and safe carrier. To date, polymeric and viral vectors have been tested for evaluation of gene therapy approaches. Although viral vectors had high efficiency in transduction, the intrinsic problems of viral vectors such as oncogenesis and immunogenesis have strictly limited their application to GLP-1 gene therapy. Due to the slow progression of diabetes, toxic carriers such as viral vectors will not be used for GLP-1 diabetes gene therapy as current forms. Instead, polymeric vectors have some advantages, compared with viral vectors. Currently, the transfection efficiency of polymeric carriers may not be enough for clinical application. However, their safety profile suggests that active research in this field may produce a useful carrier in a near future. The second approach is to use hydrogel or microsphere as a depot for controlled release of GLP-1. The stability of GLP-1 in the depot should be confirmed and the safety should be proved. In terms of safety, ReGel and PLGA microspheres have been evaluated in preclinical and clinical trials, suggesting their applications in clinics in future. The third approach is to modify for protection of the GLP-1 peptide from the degradation by enzymes. PEGylation, albumin conjugation, or the insertion of disulfide bond have been evaluated. The modification increased the half-life as well as solubility and stability of the peptide. The last approach is to use with inhibitors against DPP-IV. Some inhibitors are commercially available for clinical use. It has the ability to inactivate DPP-IV, resulting in the prolonged half-life and activity of produced incretin hormones. However, DPP-IV itself cannot directly cause normoglycemia with no effect on gastric emptying and the improvement to complications. Also, it should be considered the durability and efficacy for the treatment of diabetes. In contrast to DPP-IV inhibitors, the described approaches have high efficacy and lots of incretin effects via single administration than DPP-IV inhibitors. However, to apply in clinical field, these systems must be considered safety, stability, and more long-lasting action to appear the therapeutic effects with no toxicity in patient. Therefore, by combining the complementary characteristics of approaches mentioned in this paper and DPP-IV inhibitors, it should be possible to overcome the limitations associated with each.

In trade of recent decade based on above-mentioned strategies, most studies have been focused on improvement of the pharmacokinetics of GLP-1 or exendin-4. However, besides the induction of exogenous incretins, efforts to increase endogenous GLP-1 production are needed to be parallel for the treatment of diabetes such as a-glycosidase inhibitor [6, 82] or miglitol [83].

**Figure 2:**
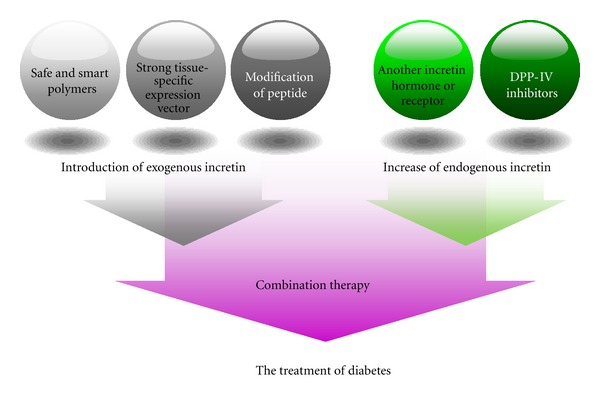
Potentiation therapy based on incretin for the treatment of diabetes.

At this point, more investigations remained to be explored. For example, it is that activator, inhibitor, or siRNA capable of activation or inactivation of action mechanism of GLP-1 and its receptor or target of another kind of receptor such as GLP-2 receptor. At the same time, the sensitivity of these treatments to patients has to be considered for personalized diagnosis and therapy due to the difference against GLP-1 receptor responsiveness and sensitivity in each person. Finally, diabetic treatment will need combination therapy with other existing treatment to induce an increase of both endogenous and exogenous incretin hormones for sufficient therapeutic efficacies (Figure 2). In this sense, polymeric-based on incretin approaches may serve as intriguing tools to generate more therapeutic GLP-1 or exendin-4 for diabetes. With all these efforts, therapeutic applications of incretin will be realized as the positive impact with little side-effects in a near future.

## Figures and Tables

**Table 1:** Classification of expression vector systems of GLP-1 or related mimetic gene with various polymer carriers.

Delivery carriers	Therapeutic material	Expression vector system	Characterizations	Applications	*In vivo* injection route	Glycemic effect period	Diabetes type	Ref
PEI	GLP-1	p*β*-GLP-1	GLP-1 gene driven via chicken *β*-actin promoter/enhancer	*In vitro *and* in vivo (ZDF rats) *	i.v. (1x)	14 days	T2D	[29]
	GLP-1	pSIGLP-1/NFkB	Enhanced nuclear import by insertion of NF*κ*B binding site	*In vitro *and* in vivo *(DIO mice)	i.v. (1x)	21 days	T2D	[30]
	GLP-1	TSTA-GLP-1	GLP-1 expressed by TSTA system	*In vitro*	—	—		[31]

ABP	GLP-1	TSTA-SP-GLP-1	GLP-1 gene driven by TSTA with SP using bioreducible polymer	*In vitro*	—	—		[33]
	Exendin-4	TSTA-SP-exendin-4	Exendin-4 gene delivery by TSTA with SP using bioreducible polymer	*In vitro and in vivo *(DIO mice)	i.v. (1x)	12 days	T2D	—

Chitosan	GLP-1	pVax1-GLP-1	Plasmid DNA TNC with GLP-1 expressed by CMV promoter	*In vitro and in vivo *(db/db mice)	i.m. or s.c. (7x)	23 or 29 days	T2D	[34]

Elctroporation	GLP-1	GLP-1-IgG-Fc/VRew	plasmid encoding active human GLP-1 and mouse IgG1 heavy chain constant regions (Fc)	*In vivo *(db/db mice)	i.m. (2x)	12 weeks	T2D	[35]

*β*-actin: chicken beta-actin promoter.

NF*κ*B: nuclear factor *κ*B.

TSTA system: two-step transcription amplification system.

SP: secretion signal peptide.

TNC: therapeutic nanocomplex.

CMV: cytomegalovirus promoter.

i.v., i.m., or s.c.: intravenous, intramuscular, or subcutaneous injection.

T2D: type 2 diabetes.

**Table 2:** Classification of expression vector system of GLP-1 or related mimetic gene in viral vectors.

Delivery carriers	Therapeutic material	Expression vector system	Characterizations	Applications	*In vivo* injection route	Glycemic effect period	Diabetes type	Ref
Ad	GLP-1	Ad-CUbiEX4GLP-1Gly^8^	GLP-1 linked to Ex4 leader sequence with CMV enhancer/ubiquitin promoter and ubiquitin B intron (CUbi) in Ad gene	*In vitro and in vivo *(db/db mice or ZDF rats)	i.v. (1x)	6 weeks	T2D	[36]
	GLP-1	Ad2-CMV-GLP-1	GLP-1 driven by CMV promoter/*β*-globin/IgG chimeric intron/albumin leder sequence in Ad2 gene	In vitro and in vivo (NOD/SCID or NOD mice)	i.v. (1x)	30 dyas or 12 months	T1D	[37]
				*In vivo *(ob/ob mice)	i.v. (1x)	8 weeks	T2D	[37]
	Exendin-4	Helper-dependent Ad-CMV-exendin-4	Exendin-4 expressed by CMV promoter/mouse IgG *κ* light chain leader/furin cleavage site in helper-dependent Ad gene	*In vitro and in vivo *(DIO mice)	i.v. (1x)	15 weeks	T2D	[38]

Ad or AAV	GLP-1	AAV- or Ad-IL-GLP-1	GLP-1 driven by CMV promoter/*β*-globin intron/insulin leader sequence in AAV or Ad gene	*In vitro and in vivo *(db/db mice or ZDF rats)	i.v. (1x)	3 weeks	T2D	[39]

AAV	GLP-1	dsAAV-MIP-GLP-1	dsAAV8 vector containing GLP-1 gene expressed by mouse insulin-II promoter/proglucagon signal sequence	*In vitro and in vivo *(Balb/c)	i.p. (1x)	3 weeks	T1D	[40]
	GLP-1	dsAAV-CB-GLP-1	dsAAV2 containing GLP-1 expressed by CB promoter/Ig k-chain leader sequence/HA epitope tag/a furin protease recognition sequence	*In vitro and in vivo *(db/db mice)	i.v. (1x)	4 months	T2D	[41]

Ad: adenovirus.

AAV: adeno-associated virus.

dsAAV: double strand adeno-associated virus.

CMV promoter: cytomegalovirus promoter.

i.v., or i.p.: intravenous, or intraperitoneal injection.

CB promoter: CMV enhancer/chicken *β*-actin promoter.

T1D or T2D: type 1 diabetes or type 2 diabetes.

**Table 3:** Classification of GLP-1 or GLP-1-related mimetic peptides modified with polymers via chemical conjugation.

Delivery carriers	Therapeutic material	Modified peptide	Characterizations	Applications	*In vivo* injection route	Glycemic effect period	Diabetes type	Ref
ReGel	GLP-1	ZnGLP-1 in ReGel	Sustained release of zinc-complexed GLP-1 formulated in ReGel (triblocopolymers)	*In vitro and in vivo *(ZDF rats)	s.c. (1x)	2 weeks	T2D	[42]
VAP and alginate	GLP-1	VAPG	Rat islets and VAPG macroencapsulated in alginate	*In vitro*	—	Insulinotropic effect		[43]
PLGA	Exendin-4	Palmityl-acylated exendin-4	palmityl-acylated exendin-4 adsorbed onto porous PLGA microparticle	*In vitro and in vivo *(db/db mice)	Pulmonary (1x)	5 days	T2D	[44]

PEG	GLP-1	mPEG-Lys-GLP-1	PEGylated GLP-1 peptide with 2 k mPEG at free amine function groups in C-terminal (Lys^26^ and Lys^34^)	*In vitro* and *in vivo* (SD rats)	i.v. or s.c. (1x)	Pharmaco-kinetics (2 h)	T2D	[45]
	Exendin-4 and glucagon agonist	Dual-acting hybrid peptide with several mutations	PEGylated either GLP-1 or exendin-4 and a glucagon receptor antagonist peptide with 22 or 43 kDa PEG	*In vitro* and *in vivo* (Wistar rats)	s.c. (1x)	17 h (IPTGT)	T2D	[46]
Albumin	GLP-1	CJC-1131	GLP-1 with a short covalent reactive chemical linker that interacts with a specific cysteine residue in the albumin molecule	*In vitro and in vivo *(db/db mice)	i.p. or s.c. (2x daily for 4 weeks)	6 weeks	T2D	[47]
Albumin-PEG	Exendin-4	HAS-PEG-exendin-4	Exendin-4-conjugated with human serum albumin via heterobifunctional PEG	*In vitro and in vivo *(db/db mice)	i.p. (1x)	120 h	T2D	[48]

—	GLP-1	GLP-1 containing disulfide bond	Cysteine residue mutated at position of 10, 23, and 30 in wild-type GLP-1	*In vivo *(SD or ZDF rats)	s.c. (7x)	35 days	T2D	[49]
—	GLP-1 or Exexdin-4	GLP-1 or exendin-4 containing additional disulfide bond and glycine residues	Several cysteine residues mutations in native GLP-1 and insertion of one or more glycine residues at C-terminal	*In vitro and in vivo *(SD or ZDF rats)	s.c. (7x)	35 days	T2D	[50]

CJC-1131: drug affinity complex drug affinity complex (DAC).

VAPG: poly(N-vinylpyrrolidone-co-acrylic acid-g-PEG) (VAP)-GLP-1.

i.v., s.c., or i.p.: intravenous, subcutaneous, or intraperitoneal injection.

IPGTT: intraperitoneal glucose tolerance test.

T2D: type 2 diabetes.
